# Growth and cognition over eight years in three sites of the MAL-ED cohort study

**DOI:** 10.1371/journal.pgph.0007217

**Published:** 2026-09-11

**Authors:** Amber R. Huggins, Rebecca J. Scharf, Elizabeth T. Rogawski McQuade, Erling Svensen, Angelina Maphula, Eliwaza Bayo, Ladislaus Blacy, Paula Pamplona E. de Souza, Hilda Costa, Eric R. Houpt, Pascal O. Bessong, Estomih Mduma, Aldo A. M. Lima, Mark D. DeBoer, Richard L. Guerrant

**Affiliations:** 1 Department of Public Health Sciences, University of Virginia, Charlottesville, Virginia, United States of America; 2 Department of Pediatrics, University of Virginia, Charlottesville, Virginia, United States of America; 3 Department of Epidemiology, Emory University, Atlanta, Georgia, United States of America; 4 Haukeland University Hospital, Bergen, Norway; 5 University of Venda, Thohoyandou, South Africa; 6 Haydom Lutheran Hospital, Haydom, Tanzania; 7 Universidade Federal do Ceara, Fortaleza, Brazil; 8 Division of Infectious Diseases and International Health, University of Virginia, Charlottesville, Virginia, United States of America; PLOS: Public Library of Science, UNITED STATES OF AMERICA

## Abstract

The objective of this study was to examine if height at 2 years, 5 years, and 6–8 years of age was related to school-age cognitive development in low resource settings. Some studies report improved scores in those who catch up, while others found no improvement. We hypothesize that persistent stunting will be negatively associated with cognition and that children who experienced catch-up growth would show improved cognition.Follow-up anthropometry and cognitive assessments at 6–8 years were completed in the Brazil, South Africa, and Tanzania MAL-ED cohorts. Semantic and phonemic fluency and reasoning skills were assessed with the Developmental NEuroPSYchological test and Raven Coloured Matrices, respectively. Stunting at 2, 5, and 6–8 years was used to categorize growth into persistently stunted, early catch-up, late catch-up, and never stunted. Multivariable linear regression was used to estimate relationships between linear growth, stunting, and catch-up growth and 6–8-year-old cognition.Stunting prevalence decreased with each follow-up assessment from 40.1% at 2 years to 13.4% at 6–8 years. Height at each age was negatively associated with cognition (Semantic Fluency: 0.2 z-scores, 95% CI: 0.1, 0.3). Persistent stunting was associated with decreased cognitive scores at 6–8 years (Semantic Fluency: -0.4 z-scores, 95% CI: -0.8, -0.03). Children with catch-up growth, both early (Semantic Fluency: -0.4 z-scores, 95% CI: -0.6, -0.1) and late (Semantic Fluency: -0.4 z-scores, 95% CI: -0.8, -0.1), did not have improved scores compared to those persistently stuntedLinear growth at 2 years remains an important indicator of cognitive development in late childhood. Efforts to reduce early stunting may continue as catch-up growth is not always associated with improved cognitive functioning.

## Introduction

Early childhood cognition is an important indicator of future career and learning potential [1]. Cognition among children in low- and middle-income countries (LMICs) is affected by early life exposures prior to 2 years of age, such as breastfeeding, nutrition, enteric infections, maternal education, and factors related to socioeconomic status [2–5].

Cognitive function is difficult to measure among infants and young children when exposures to poor resource environments may have the biggest impact. Assessments of physical growth in early childhood have commonly been used as a surrogate for child development when cognitive functioning is difficult to assess [6]. Measuring height rather than cognition in early to mid-childhood could be a simpler and more cost-effective way of predicting cognitive development. Linear growth at 2 years is a commonly used metric to predict cognitive functioning later in childhood due to a previously established relationship and ease of collection [6].

Stunting is characterized as having a height-for-age z-score (HAZ) below –2 [7]. In 2020, 22% of preschool-aged children (0–4 years old) were stunted across the world [8]. Within specific regions, 11% of Latin American and Caribbean children and 32% of Sub-Saharan African children were stunted [8]. The countries included in the current analysis, Brazil, South Africa, and Tanzania, have 6%, 23%, and 32% of children ages 0–4 years of age with stunting, respectively [8]. Environmental factors, such as enteric infections, have been associated with malnutrition and growth deficits [4,9], which may also contribute to cognitive deficits. These challenges further contribute to children being unable to experience catch-up growth, in which children have compensatory growth to normalize height after previous stunting. Children who experience stunting can potentially see long-term challenges in their school performance, behavior, and psychological well-being [10]. Stunting has also been associated with an increased risk of mortality and morbidity as well as suboptimal cognitive development and economic productivity [11,12].

The first 2 years of life are key in child growth and development, but findings on relevance of catch-up growth later in childhood to cognitive function are conflicting [13]. Some studies have reported catch-up growth helped prevent additional cognitive deficits [13,14], while other studies found no associations between growth after early childhood and cognitive scores [15]. Assessing cognitive functioning during 6–8 years of age, at the beginning of school-age, captures a significant time period in the formation of childhood learning.

Our goal in the current analysis was to examine the relationship between stunting at several time points in childhood and school-age cognitive scores in this longitudinal sample of children from three settings around the world in which data points on stunting were available from multiple time periods from birth to early school-age. We hypothesized that persistent stunting would be negatively associated with cognition and that children who experienced catch-up growth would show improved cognition. This paper aims to use comprehensive, longitudinal data to enhance the literature on stunting and catch-up growth and its relation to cognition.

## Methods

### Ethics statement

The study was approved by the Institutional Review Board for Health Sciences Research, University of Virginia, USA (HSR IRB #14595) as well as the respective governmental, local institutional, and collaborating institutional ethical review boards at each site: Committee for Ethics in Research, Universidade Federal do Ceará; National Ethical Research Committee, Health Ministry, Council of National Health (Brazil); Health, Safety and Research Ethics Committee, University of Venda; Department of Health and Social Development, Limpopo Provincial Government (South Africa); Medical Research Coordinating Committee, National Institute for Medical Research; Chief Medical Officer, Ministry of Health and Social Welfare (Tanzania). Informed written consent was obtained from the parent or guardian of each participating child on their behalf. Clinical Trial Registration was NCT02441426.

### Study design

Previous literature on the original MAL-ED study [2], has described baseline characteristics [16–18], and outcomes [19]. The original study collected data on enteric infections, nutrition, environmental characteristics, growth, and cognitive outcomes on children from eight low-resource settings from birth to 2 years. The MAL-ED study enrolled children within 17 days of birth in research sites that had high prevalence of malnutrition and enteric disease. Infants who were born prematurely (<37 weeks gestation), low birthweight (<1500 grams), twin gestations, or to mothers <16 years of age were excluded [2,6]. Additional anthropometric and cognitive data was collected at 6–8 years (in the study also referred to as school-age), from the research sites in Fortaleza, Brazil, Venda, South Africa, and Haydom, Tanzania. These three research sites continued to keep in contact with the study participants and had the capacity and availability to collect further data at this time period. For the current analysis, we evaluated for relationships between early childhood growth and 6–8-year-old cognitive skills using participant data from November 2009 to September 2019.

### Predictors

Field workers measured length (cm) using measuring boards at enrolment and 24 months. Height (cm) was measured using a stadiometer at the 5- and 6–8-year assessments [2,6]. Scales (Seca 354 or Detecto 8440) were calibrated weekly using standard weights. A portion of all anthropometry measurements weekly were verified by senior study personnel to ensure accuracy. The length and height measurements were then converted into z-scores using the WHO Growth Standards [20].

### Covariates

Covariates in this study included the child’s age (years), enrolment weight (measured within 17 days of birth as a proxy for birthweight, grams converted to z-scores), sex, breastfeeding status (percent days breastfed in the first 6 months of life), research site, maternal education (years), maternal height (cm), and a measure of socioeconomic status including water, assets, maternal education, and family income (Water/sanitation, Assets, Maternal education, and Income [WAMI] score) [21].

### Outcomes

At 6–8 years of age, cognitive assessments were performed to assess the participants’ semantic and phonemic fluency and reasoning skills. Further information on overall methods for cognitive assessments in the MAL-ED study are described in previous publications [2,6,22–26]. These assessments were chosen as they are relatively language and culturally facile, so that they could be used across continents, cultures and languages. To test verbal fluency among the 6–8-year-old children, field workers administered a subset of the Developmental NEuroPSYchological Assessment (NEPSY) [27]. Semantic fluency was determined by asking the children, in one minute, to list as many animals as possible, and then to list as many foods as possible in one minute. The phonemic fluency test requested children to say as many words that began with S in one minute, and then as many words that began with F in one minute. This assessment was piloted on children from the local area who were not participating in the study to assess for distribution of scores and feasibility. Researchers from this experience from the Brazil site of the MAL-ED Study found limitations in verbal fluency in children exposed to early life diarrhea and malnutrition [23,24].

The Raven Coloured Progressive Matrices (RCM) assess reasoning skills from age 5 years to 99 years [25,26]. This test is thought to be more generalizable across cultures and in populations that lack formal education because it is visually based and does not require language to complete [25]. The generalizability and familiarity of the RCM tests to the research sites made it appealing to use as a cognitive measure in the 6–8-year-old children. Additionally, all three research sites had experience using the assessment as it was given to the mothers of the children in the MAL-ED cohort [22,28]. Trained administrators guided the children through the assessment, stopping when a child had 5 incorrect answers consecutively [22]. For both NEPSY and RCM tests, raw scores were converted into z-scores to compare between participants and across sites.

Field workers at all three research sites received training on cognitive assessment administration throughout the MAL-ED Study, including several in-person trainings with developmental psychologists [22] and weekly cognitive team research study calls throughout the first five years of the study. For the assessments, training refresher was completed for the RCM assessment and field team supervisors monitored about 10% of administrations, and all forms were checked and double entered to ensure quality of data.

### Analysis

All variables were assessed for normality. To estimate the associations between height at each time point (HAZ or length-for-age z-scores [LAZ] at age 2 years) and 6–8 year cognitive scores (NEPSY and RCM z-scores), we first performed linear regression, adjusting for potential confounders, including child’s age, enrolment weight, sex, breastfeeding status, research site, maternal education, maternal height, and socioeconomic status (WAMI score). Research study site was used as a fixed effect in all models to account for clustering and variability by site. Due to sample size constraints, as well as not wanting to compare cognitive scores across sites, only within- models were not run individually by site.

Next, we dichotomized the 2-, 5-, and 6–8-year-old LAZ and HAZ variables into ‘stunted’ (LAZ/HAZ < -2) and ‘not stunted’ (LAZ/HAZ ≥ -2). Using the same 6–8-year-old cognitive scores as the outcomes, we adjusted for the covariates above in a multivariable linear regression.

To explore the relationship between catch-up growth and cognition, we categorized children into four groups: ‘never stunted’ (not stunted at 2, 5, or 6–8 years), ‘persistently stunted’ (stunted at all three assessments), ‘late catch-up growth’ (stunted at 2 and 5 years but not at 6–8 years), and ‘early catch-up growth’ (stunted at 2 years but not at 5 or 6–8 years). There were five children who were not stunted at 2 or 5 years but experienced stunting at 6–8 years, one child who was stunted at 2 and 6–8 years, but not at 5 years, one child who was stunted at 5 and 6–8 years but not at 2, and six children who were not stunted at 2 or 6–8 years but stunted at 5 years. These 13 children (2.8%) were excluded from the analysis, which was completed on 97.2% of the sample. We estimated the associations between these four categories and semantic fluency, phonemic fluency, and reasoning skills at age 6–8 years through multivariable linear regression and adjusting for the covariates above. The “never stunted” group was used as the reference. All analyses were conducted in SAS Version 9.4.

## Results

### Demographics and stunting prevalence

**Fig 1:**
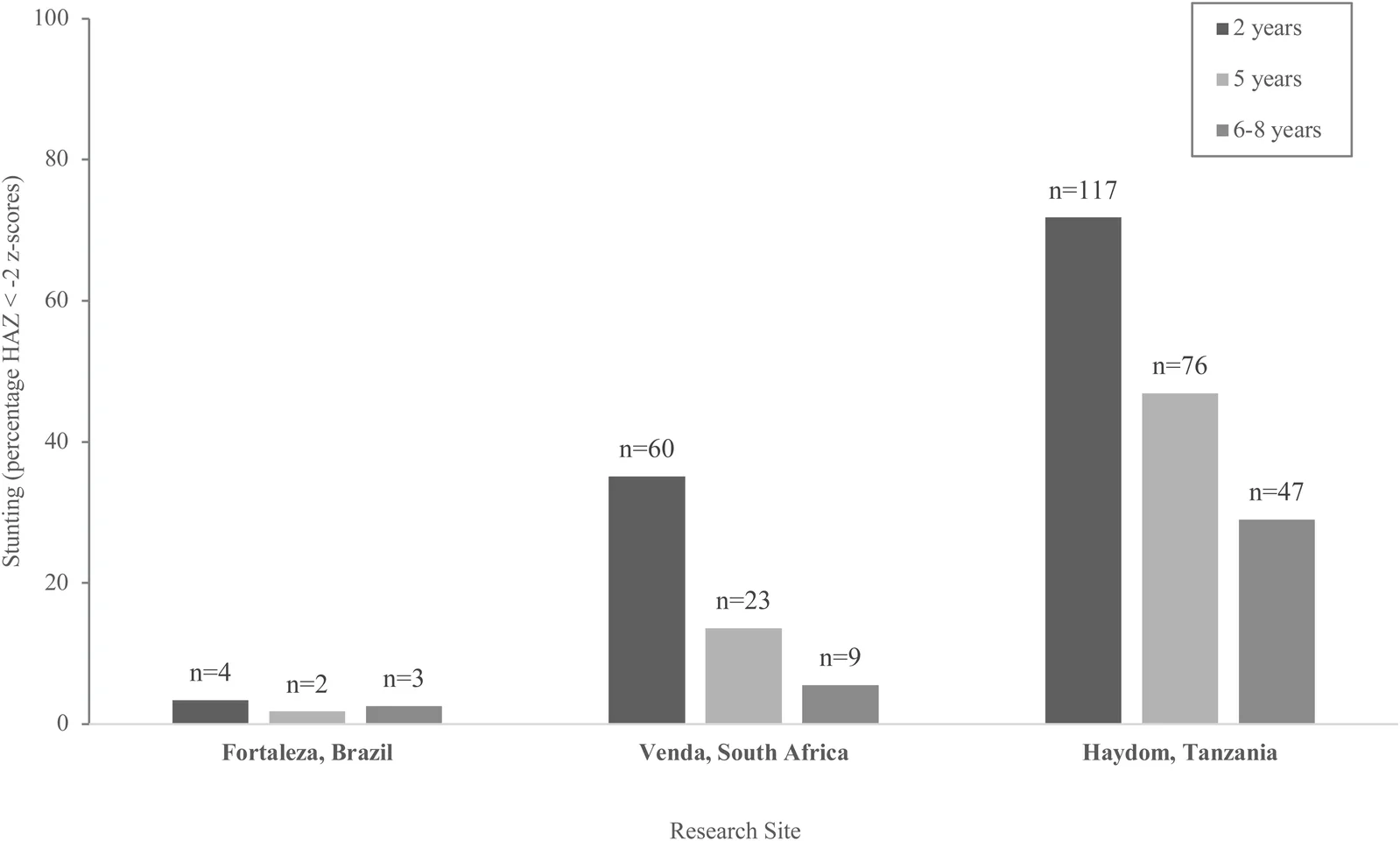
Percentage of stunting at each cognitive assessment by research site. Stunting was defined as height-for-age z-scores less than -2 z-scores. Total denominator for each year (2, 5, 6-8 years) respectively as follows: BR: n = 117, 112, 115; SA: n = 171, 169, 163; TZ: n = 163, 162, 162.

**Fig 2:**
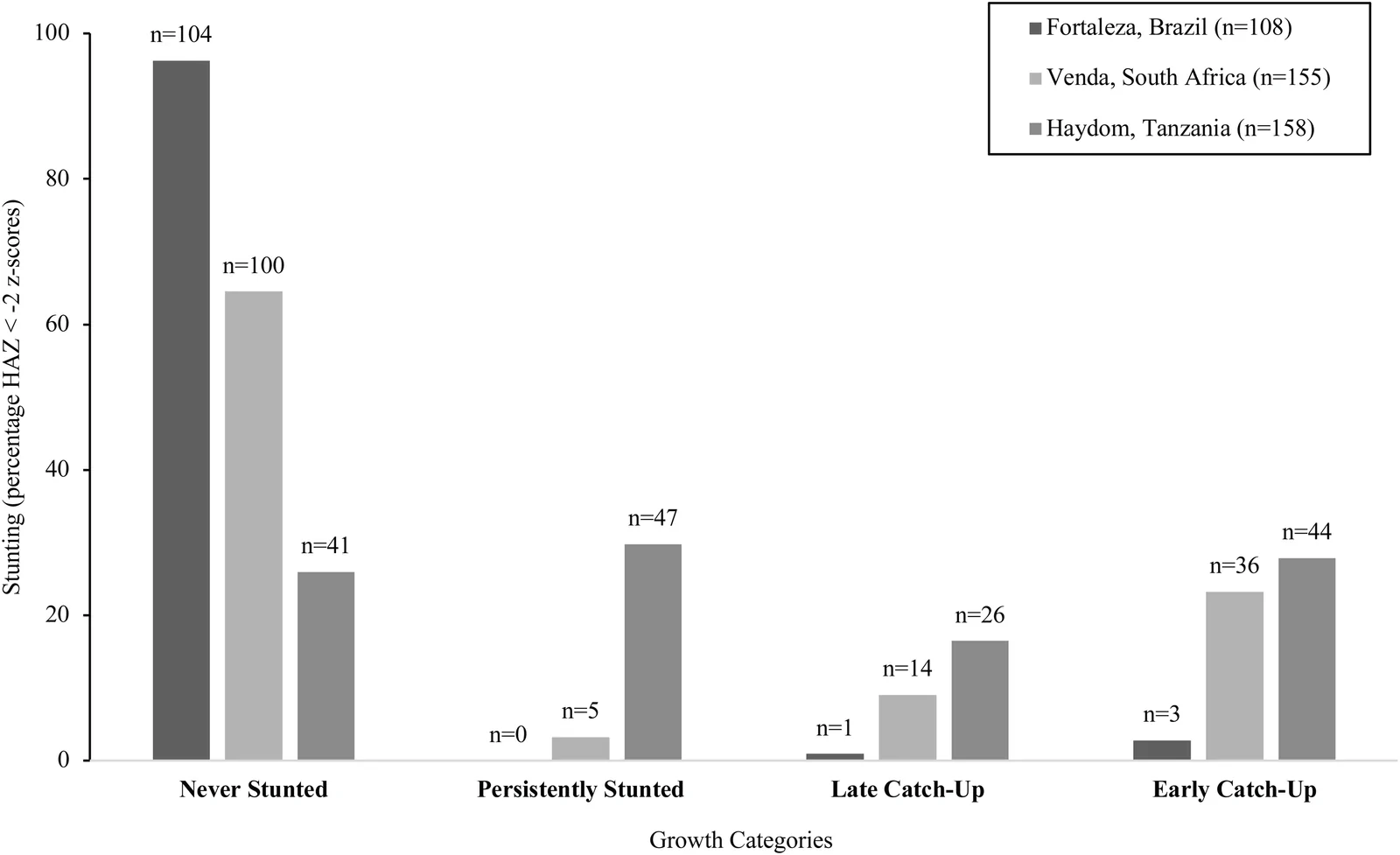
Percentage of stunting and catch-up growth by research site. Never stunted: not stunted at any assessment; Persistently stunted: stunted at all assessments; Early catch-up: stunted at 2 years but not at 5 or 6-8 years; Late catch-up: stunted at 2 and 5 years but not at 6-8 years. Denominators for each country shown in legend (total n = 421). There were 5 children who were not stunted at 2 or 5 years but experienced stunting at 6-8 years, 1 child who was stunted at 2 and 6-8 years, but not at 5 years, 1 child who was stunted at 5 and 6-8 years but not at 2, and 6 children who were not stunted at 2 or 6-8 years but stunted at 5 years. These 13 children were excluded from the analysis.

We examined data from 451 children who had anthropometry measurements, NEPSY scores, or RCM scores at 6–8 years. Table 1 summarizes the demographics of the three sites separately and in total. A previous publication compared the 451 children still in the study at school-age with the 611 children in the original study from birth to 2 years of age, and found that they had very similar characteristics [29] (see S1 Table)). Children in the Brazil site were younger on average, and the Tanzania site had lower WAMI scores on average. Stunting frequency decreased over time. Between the three sites, 40.1% (n = 181) of children were stunted at 2 years, 22.8% (n = 101) were stunted at 5 years, and 13.4% (n = 59) were stunted at 6–8 years. The children in the Tanzania research site had the highest prevalence of persistent stunting when compared to the other two research sites, with 29.0% stunted at 6–8 years (representing 79.7% of all children stunted at this time point across the 3 research sites). Fig 1 shows the distributions of stunting trajectories across each age of assessment by research site. Brazil had the highest frequency of children who were never stunted (96.3%, n = 104) (Fig 2). Of those who were stunted at 2 years, 29.5% (n = 124) of children caught up at 6–8 years. Between all three sites, more children experienced early catch-up (19.7%, n = 83) than late catch-up (9.7%, n = 41) and persistent stunting (12.4%, n = 52).

**Table 1:** Sociodemographic and anthropometry of each research site and overall.

	Fortaleza, Brazil (n = 117)	Venda, South Africa (n = 171)	Haydom, Tanzania (n = 163)	Overall (n = 451)
**Sociodemographic characteristics**	*Median (IQR)*			
Age at 6–8-year assessment	6.9 (6.4, 7.2)	8.0 (7.1, 8.2)	7.7 (7.3, 8.0)	7.5 (7.0, 8.0)
Age at 5-year assessment	5.0 (5.0, 5.1)	5.0 (5.0, 5.0)	5.0 (5.0, 5.0)	5.0 (5.0, 5.0)
Age at 24-month assessment	2.0 (2.0, 2.0)	2.0 (2.0, 2.0)	2.0 (2.0, 2.2)	2.0 (2.0, 2.0)
Female sex (n; %)	53 (45.3)	84 (49.1)	82 (50.3)	219 (49.0)
Percent days exclusively breastfed < 6 mo.	0.5 (0.3, 0.7)	0.2 (0.1, 0.3)	0.3 (0.2, 0.4)	0.3 (0.2, 0.5)
Mean WAMI Score	0.8 (0.8, 0.9)	0.8 (0.7, 0.9)	0.2 (0.1, 0.3)	0.7 (0.3, 0.8)
Maternal height (cm)	155.0 (150.0, 160.0)	158.0 (155.0, 162.0)	156.5 (152.0, 160.0)	157.0 (152.0, 161.0)
Maternal education (years)	9 (7, 12)	11 (9, 12)	7 (3, 7)	8 (7, 11)
Income (dollar)	345.8 (292.1, 412.8)	246.5 (164.4, 396.8)	21.0 (13.2, 39.0)	186.0 (32.6, 344.9)
**Anthropometry**				
Enrolment weight-for-age z-score	−0.2 (−0.8, 0.5)	−0.4 (−1.0, 0.1)	−0.02 (−0.6, 0.6)	−0.2 (−0.8, 0.4)
2-year length-for-age z-score	−0.03 (−0.9, 0.7)	−1.6 (−2.5, −1.1)	−2.6 (−3.2, −2.0)	−1.7 (−2.6, −0.7)
5-year height-for-age z-score	−0.1 (−0.8, 0.4)	−1.1 (−1.6, −0.4)	−1.9 (−2.5, −1.2)	−1.2 (−1.9, −0.4)
6–8-year height-for-age z-score	−0.1 (−0.7, 0.5)	−0.1 (−0.8, 0.5)	−1.6 (−2.2, −1.0)	−0.7 (−1.6, 0.2)

### Associations between height and cognitive scores

Each 1 SD increase in LAZ at 2 years was associated with higher scores for semantic fluency (0.2 z-scores, 95% CI: 0.1, 0.3) and reasoning skills (0.1 z-scores, 95% CI: 0.01, 0.2) (Table 2). There was a similar trend for phonemic fluency, but it was not statistically significant. Estimates were similar in magnitude for LAZ at 5- and 6–8-years-old and statistically significant.

**Table 2:** Associations between linear growth and cognition.

	Semantic Fluency	Phonemic Fluency	Reasoning Skills
	Words/Animals in a minute from NEPSY	Words beginning with S & F in a minute from NEPSY	Raven Coloured Progressive matrices
**Linear Growth (per z-score increase)**	*Estimate (CL) P-value*		
LAZ at 2 years	0.2 (0.1, 0.3) 0.001	0.1 (−0.02, 0.2) 0.12	0.1 (0.01, 0.2) 0.03
HAZ at 5 years	0.2 (0.1, 0.3) 0.002	0.1 (0.03, 0.2) 0.01	0.2 (0.1, 0.3) 0.002
HAZ at 6–8 years	0.2 (0.1, 0.3) 0.001	0.2 (0.1, 0.3) < 0.001	0.1 (0.03, 0.2) 0.01
**Stunting at Each Year (vs. Not Stunted)**			
2 years	−0.3 (−0.6, −0.1) 0.01	−0.1 (−0.3, 0.1) 0.30	−0.3 (−0.5, −0.1) 0.02
5 years	−0.2 (−0.5, 0.02) 0.07	−0.1 (−0.4, 0.1) 0.24	−0.2 (−0.5, 0.03) 0.09
6–8 years	−0.2 (−0.5, 0.1) 0.12	−0.2 (−0.5, 0.1) 0.14	−0.2 (−0.4, 0.1) 0.29
**Growth and Catch-up (vs Never Stunted)**			
Persistent Stunting	−0.4 (−0.8, −0.03) 0.04	−0.2 (−0.5, 0.2) 0.31	−0.4 (−0.7, −0.01) 0.05
Late Catch-Up	−0.4 (−0.8, −0.1) 0.02	−0.2 (−0.6, 0.1) 0.20	−0.4 (−0.8, −0.1) 0.02
Early Catch-Up	−0.4 (−0.6, −0.1) 0.01	−0.1 (−0.4, 0.2) 0.50	−0.3 (−0.6, −0.04) 0.02

Stunting as defined by HAZ less than -2. Persistently stunted: stunted at all assessments; Early catch-up: stunted at 2 years but not at 5 or 6–8 years; Late catch-up: stunted at 2 and 5 years but not at 6–8 years. Scores reflect estimates of differences in cognitive scores between growth categories, adjusted for child’s age (years), enrolment weight, sex, breastfeeding status, research site, maternal education, maternal height, and socioeconomic status (WAMI score) through linear regression.

**Fig 3:**
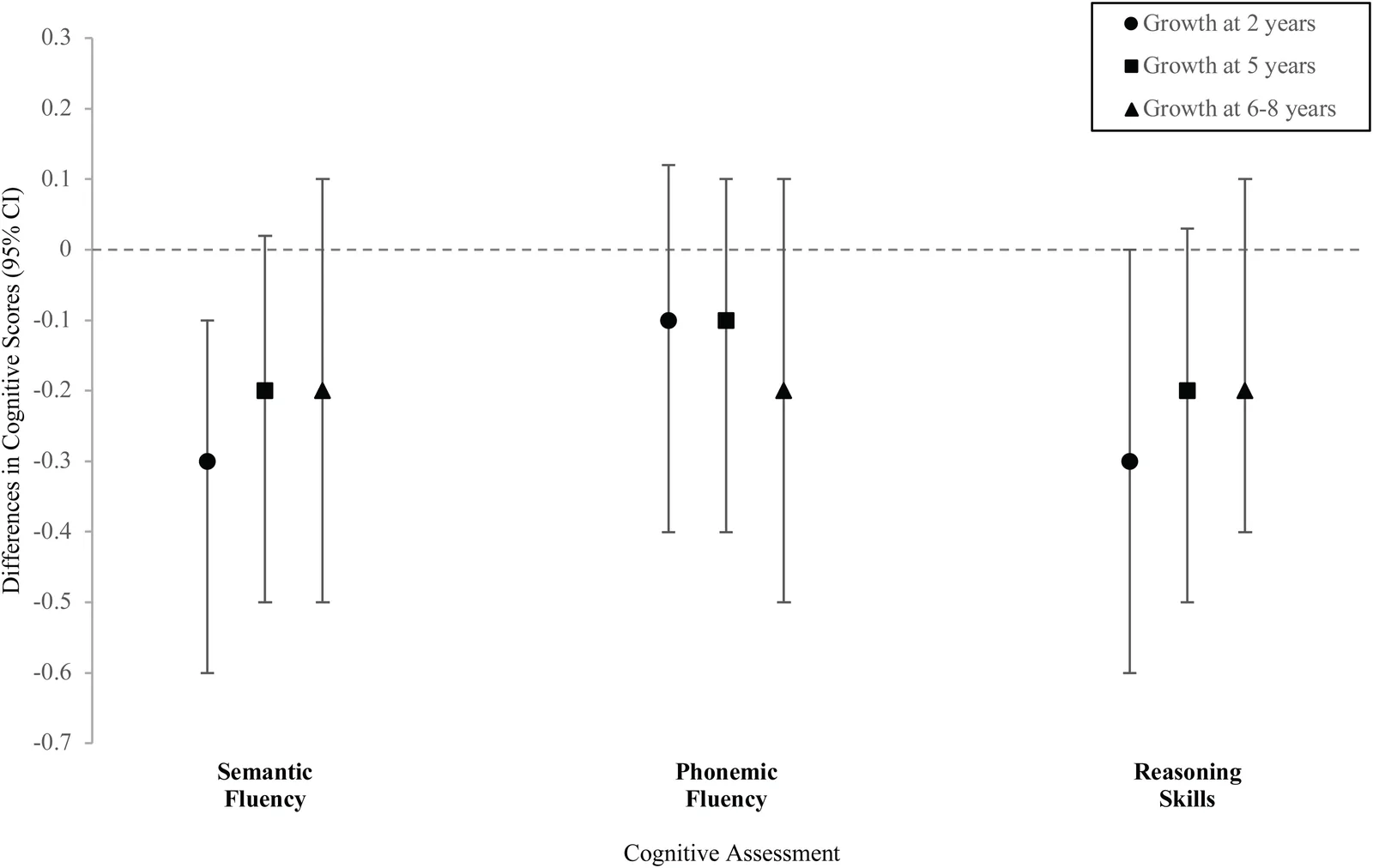
Associations between dichotomous growth at 2, 5, and 6-8 years and school-aged cognitive scores when compared to children who were never stunted. Dichotomous stunting (yes/no), as defined by HAZ less than -2, at 2, 5, and 6-8 years. Scores reflect estimates of differences in cognitive scores between growth categories, adjusted for child’s age (years), enrolment weight, sex, breastfeeding status, research site, maternal education, maternal height, and socioeconomic status (WAMI score) through linear regression.

The associations between dichotomous stunting and cognition at each age assessment are summarized in Fig 3. These relationships were most noted at 2 years, at which point stunting conferred slightly lower scores in semantic fluency (-0.3 z-scores, 95% CI: -0.6, -0.1) and reasoning skills (-0.3 z-scores, 95% CI: -0.5, -0.1). While similar magnitude of deficits was noted with stunting at 5 and 6–8 years, these associations were not statistically significant.

### Associations with catch-up growth

**Fig 4:**
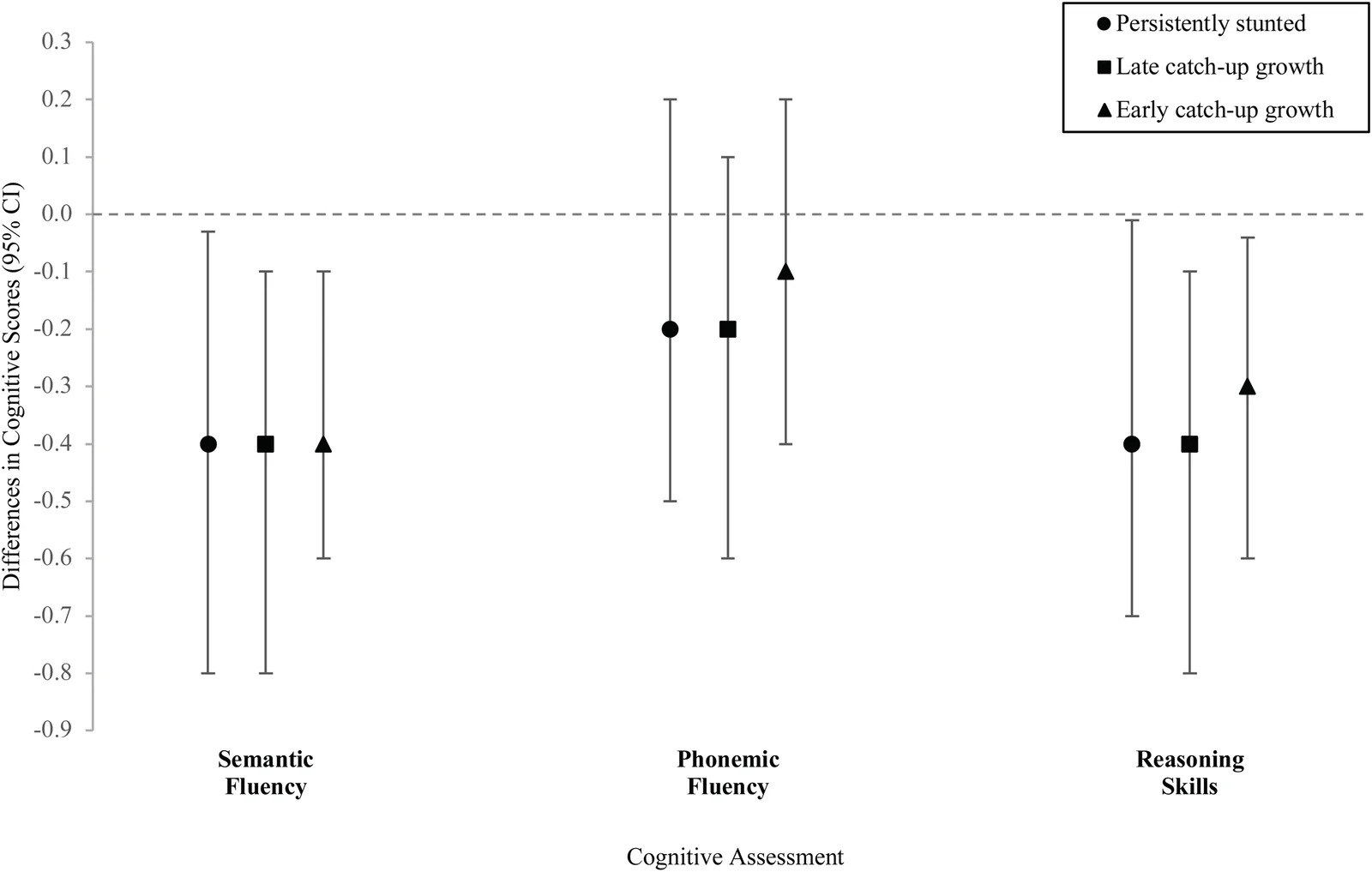
Associations between catch-up growth and school-aged cognitive scores when compared to children who were never stunted. Stunting as defined by HAZ less than -2. Persistently stunted: stunted at all assessments; Early catch-up: stunted at 2 years but not at 5 or 6-8 years; Late catch-up: stunted at 2 and 5 years but not at 6-8 years. Scores reflect estimates of differences in cognitive scores between growth categories, adjusted for child’s age (years), enrolment weight, sex, breastfeeding status, research site, maternal education, maternal height, and socioeconomic status (WAMI score) through linear regression.

The associations between the longitudinal assessment of stunting and poor cognition in the setting of catch-up growth can be found in Fig 4. Associations were similar between stunting categories for each cognitive assessment. Compared to those who were never stunted, those who were persistently stunted had significantly lower scores for semantic fluency (-0.4 z-scores, 95% CI: -0.8, -0.03). Children who experienced later catch-up growth at 6–8 years also had significantly lower scores for semantic fluency (-0.4 z-scores, 95% CI: -0.8, -0.1) and reasoning skills (-0.4 z-scores, 95% CI: -0.8, -0.1) when compared to those who were never stunted (Fig 4). Similar negative associations were also seen for semantic fluency (-0.4 z-scores, 95% CI: -0.6, -0.1) and reasoning skills (-0.3 z-scores, 95% CI: -0.6, -0.04) in children who experienced early catch-up growth at 5 years and remained caught up at 6–8 years (Fig 4). Associations between phonemic fluency and each of the growth categories showed similar associations, but they did not reach statistical significance (Fig 4).

## Discussion

Across all three cognitive assessments, we found consistent relationships between dichotomous stunting at each age and lower cognitive scores. (Fig 3). These findings support our hypothesis that stunting is negatively associated with cognition. We also found negative associations with cognitive scores across all three 6–8-year-old outcomes for children who experienced catch-up growth compared to never stunted children (Fig 4). Children who experienced catch-up growth, both early and late, had similar associations as children who were always stunted when compared to children who were never stunted. This refutes our hypothesis that catch-up growth would ameliorate poor cognitive scores. Stunting at 2 years exhibited similar overall associations to persistent stunting, early catch-up, and late catch-up growth. This supports the use of growth at 2 years as a predictor of cognition in late childhood in settings of malnutrition and enteric disease.

While attained growth in childhood is not a perfect surrogate for cognition, measuring and collecting growth data can be more efficient in low resource settings. Testing a child’s cognitive capabilities through evaluations at various ages requires time and resources to train field workers. Growth measurements are more straightforward and cognitive performance is easier to assess at later ages [6]. While growth and cognition have shared determinants, there may be unique mechanisms for effects on each outcome. In a 2019 meta-analysis of early childhood interventions on linear growth and cognitive development, Prado et al. concluded that linear growth and cognitive development share some risk factors including nutrition [30]. However, after analyzing the effects of interventions on linear growth and cognitive scores, they found that improvements in linear growth were not consistent with improvements in cognition [30].

The findings from this study support existing research associating poor growth and lower cognitive scores [14,31]. Evidence on cognitive performance after catch-up growth past 2 years has yet to be established. The results from the 5-year-old MAL-ED follow-up study support the negative association between early-onset persistent stunting and cognitive scores [5]. They also reported that children who were stunted early but recovered by 5 years had significantly lower scores compared to never stunted children [5]. The findings from the present study support this and add that early stunting continues to be associated with development through at least 6–8 years of age in this observational study.

Our findings contradict other studies that reported catch-up growth may mitigate further cognitive delays [13,14,31]. A similar structure as Koshy et al. [13] was followed for the current analysis, categorizing growth into never stunted, early catch-up, late catch-up, and always stunted groups. Our study population also saw maximum stunting at 2 years, with the majority experiencing catch-up growth by the third assessment. They found that children who experienced catch-up growth by age 9 years after stunting at age 2 years scored higher on cognitive tests when compared to children who were never stunted [13]. They reported that scores of children who caught up were higher than the scores of persistently stunted children [13]. These findings contrast with the current analysis in which catch-up growth did not improve scores. Regardless of catch-up growth status, the effects of stunting at 2 years were still relevant.

Due to the similarity in the associations between the stunting and catch-up groups and stunting at 2 years, intervening as early as possible is recommended. Focus can be placed on shared risk factors for height and cognition, including enteric infections and socioeconomic status. However, Prado et al. emphasized caregiving interventions because they saw greater effects on neurodevelopmental scores than nutrition alone [30]. It is important to ensure children meet growth standards throughout their childhood, but emphasizing proper caregiving during the first 2 years of life may be more pertinent in preventing future cognitive delays. Indeed, a study randomizing children to intervention with responsive stimulation resulted in improved growth at age 2 years, which was greater than that seen among children randomized to receive intervention with micronutrient supplementation [32]. Efforts to improve children’s environments should be made to reduce the effects on cognition. Additionally, improvements in maternal health in low-resource settings must continue.

In these analyses, diarrhea episodes in the 0–2 year time period were not found to be independently related to these later outcomes, and thus were not ultimately included in these models. We did find that the inflammatory pathogen Shigella was related with later growth deficits [29,33].

Few studies have been completed with repeated measures for anthropometry and cognitive outcomes, making this study unique as there was ample data from birth through 6–8 years for three sites. In addition, this study has measures of executive function in three country sites, in addition to the reasoning measures, which have not been used in two of these settings previously. While having longitudinal data in children over 8 years is a strength of this study, it adds complexity in that social settings change over time. Over time in these study sites, access to food, education and clean water may have changed or improved, and this may account for some of the improved growth over time. It may also be that participation in an observational cohort study gave families more touchpoints with study personnel checking in and increased access to care, thus also improving outcomes over time. Study limitations include intrinsic differences between research sites. Children in the Tanzania site had received less formal schooling than the other two sites, making the process of naming words more difficult. There were variations in education, languages, and socioeconomic environments across sites. These may contribute to residual confounding even though study site, water sources, assets, maternal education, and family income were included in the models. It was not the intention of this study to compare cognitive results across sites, but rather to examine relationships between growth and cognitive outcomes among the various exposure levels within sites, and thus site was used as a fixed effect in the models, rather than running analyses by site, to prevent ethical challenges in comparing results across study cultures. In this study, enrolment weight was used as a proxy for birthweight as not all children were weighed at birth. Depending on the timing of enrolment, weight may be higher or lower than birthweight. Many infants lose around 7–10% of birthweight in the first week of life, but often return to birthweight by 2 weeks of age, and then may gain about 120–200 grams per week. Infants were enrolled between 2 and 17 days of life, thus birthweight and enrolment weight likely within 200–300 grams range. In this study, we used z-scores for weight for age in the analyses, thus accounting for this possible difference, as WAZ is based on age. Data also drove the measures used for “catch-up” growth. In this study, additional height measures were collected at 5 and 6–8 years, and thus catch-up growth was defined as having been stunted at age 2 years (having a height above 2 standard deviations below the mean) but not being stunted at age 5 or 6–8 years. Additionally, there was a smaller sample size within the ‘always stunted’ and ‘late catch-up growth’ groups, specifically in the Brazil research site, which made the ability to detect differences in the groups lower. The smaller sample size of each site may contribute to the lack of observed cognitive benefit among catch-up growth groups. We have used estimates and confidence intervals to report associations, but did not include coefficients of confounders as they are adjusted as mediators and not interpreted as causal effects [34].

These data contribute additional evidence to the literature of the importance of growth in the first two years of life in both rural and urban contexts, to the future cognitive and executive skills of school-age children. In addition, children who experienced catch-up growth after age 2, still did not do as well on cognitive assessments as their never-stunted counterparts in this small observational study across three research sites.

## Conclusion

In conclusion, we found that stunting and catch-up growth from early to late childhood is associated with lower cognitive scores in settings of malnutrition and enteric infections. Differences in effect size between catch-up groups were minimal. Stunting at 2 years was a marker of poorer cognition regardless of early or late catch-up growth in this study of 451 children followed from birth until school-age.

## Supporting information

S1 Table Comparison of characteristics between the 451 children assessed at 6–8 years of age and the 611 children in Brazil, South Africa, and Tanzania who completed the original MAL-ED study with follow-up to 2 years of age. (DOCX)

## References

[1] Ford ND, Stein AD. Risk factors affecting child cognitive development: a summary of nutrition, environment, and maternal-child interaction indicators for sub-Saharan Africa. J Dev Orig Health Dis. 2016;7(2):197–217. doi: 10.1017/S2040174415001427 26358240 PMC4800975

[2] MAL-ED Network Investigators. The MAL-ED study: a multinational and multidisciplinary approach to understand the relationship between enteric pathogens, malnutrition, gut physiology, physical growth, cognitive development, and immune responses in infants and children up to 2 years of age in resource-poor environments. Clin Infect Dis. 2014;59 Suppl 4:S193–206. doi: 10.1093/cid/ciu653 25305287

[3] Walker SP, Wachs TD, Gardner JM, Lozoff B, Wasserman GA, Pollitt E, et al. Child development: risk factors for adverse outcomes in developing countries. Lancet. 2007;369(9556):145–57. doi: 10.1016/S0140-6736(07)60076-2 17223478

[4] Sudfeld CR, McCoy DC, Danaei G, Fink G, Ezzati M, Andrews KG, et al. Linear growth and child development in low- and middle-income countries: a meta-analysis. Pediatrics. 2015;135(5):e1266–75. doi: 10.1542/peds.2014-3111 25847806

[5] Alam MA, Richard SA, Fahim SM, Mahfuz M, Nahar B, Das S, et al. Impact of early-onset persistent stunting on cognitive development at 5 years of age: Results from a multi-country cohort study. PLoS One. 2020;15(1):e0227839. doi: 10.1371/journal.pone.0227839 31978156 PMC6980491

[6] Scharf RJ, Rogawski ET, Murray-Kolb LE, Maphula A, Svensen E, Tofail F, et al. Early childhood growth and cognitive outcomes: Findings from the MAL-ED study. Matern Child Nutr. 2018;14(3):e12584. doi: 10.1111/mcn.12584 29392824 PMC6866087

[7] Organization WH. WHO child growth standards: methods and development: Length/height-for-age, weight-for-age, weight-for-length, weight-for-height and body mass index-for-age. Geneva, Switzerland: World Health Organization; 2006.

[8] Fund UNCs. The state of the world’s children 2021: On my mind – Promoting, protecting and caring for children’s mental health. New York: UNICEF; 2021.

[9] Petri WA Jr, Miller M, Binder HJ, Levine MM, Dillingham R, Guerrant RL. Enteric infections, diarrhea, and their impact on function and development. J Clin Invest. 2008;118(4):1277–90. doi: 10.1172/JCI34005 18382740 PMC2276781

[10] Prendergast AJ, Humphrey JH. The stunting syndrome in developing countries. Paediatr Int Child Health. 2014;34(4):250–65. doi: 10.1179/2046905514Y.0000000158 25310000 PMC4232245

[11] Richard SA, McCormick BJJ, Murray-Kolb LE, Lee GO, Seidman JC, Mahfuz M, et al. Enteric dysfunction and other factors associated with attained size at 5 years: MAL-ED birth cohort study findings. Am J Clin Nutr. 2019;110(1):131–8. 31127812 10.1093/ajcn/nqz004 PMC6599740

[12] Victora CG, Adair L, Fall C, Hallal PC, Martorell R, Richter L, et al. Maternal and child undernutrition: consequences for adult health and human capital. Lancet. 2008;371(9609):340–57. doi: 10.1016/S0140-6736(07)61692-4 18206223 PMC2258311

[13] Koshy B, Srinivasan M, Gopalakrishnan S, Mohan VR, Scharf R, Murray-Kolb L, et al. Are early childhood stunting and catch-up growth associated with school age cognition?-Evidence from an Indian birth cohort. PLoS One. 2022;17(3):e0264010. doi: 10.1371/journal.pone.0264010 35235588 PMC8890627

[14] Crookston BT, Schott W, Cueto S, Dearden KA, Engle P, Georgiadis A, et al. Postinfancy growth, schooling, and cognitive achievement: Young Lives. Am J Clin Nutr. 2013;98(6):1555–63. doi: 10.3945/ajcn.113.067561 24067665 PMC3831540

[15] Upadhyay RP, Hysing M, Taneja S, Kvestad I, Bhandari N, Strand TA. Linear growth between early and late childhood and cognitive outcomes at 6-9 years of age. J Pediatr. 2020;225:214–21.e3. 32473149 10.1016/j.jpeds.2020.05.043

[16] Lima AAM, Oria RB, Soares AM, Filho JQ, de Sousa F, Abreu CB. Geography, population, demography, socioeconomic, anthropometry, and environmental status in the MAL-ED cohort and case-control study sites in Fortaleza, Ceara, Brazil. Clin Infect Dis. 2014;59:S287–94. 10.1093/cid/ciu438 25305299

[17] Mduma ER, Gratz J, Patil C, Matson K, Dakay M, Liu S, et al. The etiology, risk factors, and interactions of enteric infections and malnutrition and the consequences for child health and development study (MAL-ED): description of the Tanzanian site. Clin Infect Dis. 2014;59 Suppl 4:S325–30. doi: 10.1093/cid/ciu439 25305305

[18] Bessong PO, Nyathi E, Mahopo TC, Netshandama V, MAL-ED South Africa. Development of the Dzimauli community in Vhembe District, Limpopo province of South Africa, for the MAL-ED cohort study. Clin Infect Dis. 2014;59 Suppl 4:S317–24. doi: 10.1093/cid/ciu418 25305304

[19] MAL-ED Network Investigators. Childhood stunting in relation to the pre- and postnatal environment during the first 2 years of life: The MAL-ED longitudinal birth cohort study. PLoS Med. 2017;14(10):e1002408. doi: 10.1371/journal.pmed.1002408 29069076 PMC5656304

[20] de Onis M, Onyango A, Borghi E, Siyam A, Blössner M, Lutter C, et al. Worldwide implementation of the WHO Child Growth Standards. Public Health Nutr. 2012;15(9):1603–10. doi: 10.1017/S136898001200105X 22717390

[21] Psaki SR, Seidman JC, Miller M, Gottlieb M, Bhutta ZA, Ahmed T, et al. Measuring socioeconomic status in multicountry studies: results from the eight-country MAL-ED study. Popul Health Metr. 2014;12(1):8. doi: 10.1186/1478-7954-12-8 24656134 PMC4234146

[22] Murray-Kolb LE, Rasmussen ZA, Scharf RJ, Rasheed MA, Svensen E, Seidman JC, et al. The MAL-ED cohort study: methods and lessons learned when assessing early child development and caregiving mediators in infants and young children in 8 low- and middle-income countries. Clin Infect Dis. 2014;59 Suppl 4(Suppl 4):S261–72. doi: 10.1093/cid/ciu437 25305296 PMC4204608

[23] Lima AAM, Kvalsund MP, de Souza PPE, Figueiredo ÍL, Soares AM, Mota RMS, et al. Zinc, vitamin A, and glutamine supplementation in Brazilian shantytown children at risk for diarrhea results in sex-specific improvements in verbal learning. Clinics (Sao Paulo). 2013;68(3):351–8. doi: 10.6061/clinics/2013(03)oa11 23644855 PMC3611743

[24] Patrick PD, Oriá RB, Madhavan V, Pinkerton RC, Lorntz B, Lima AAM, et al. Limitations in verbal fluency following heavy burdens of early childhood diarrhea in Brazilian shantytown children. Child Neuropsychol. 2005;11(3):233–44. doi: 10.1080/092970490911252 16036449

[25] Bingham WC, Burke HR, Murray S. Raven’s progressive matrices: construct validity. J Psychol. 1966;62(2):205–9. doi: 10.1080/00223980.1966.10543785 5938498

[26] Raven J. The Raven’s progressive matrices: change and stability over culture and time. Cogn Psychol. 2000;41(1):1–48. doi: 10.1006/cogp.1999.0735 10945921

[27] Korkman ME. Nepsy a neuropsychological test battery for children based on Luria’s investigation. J Clin Exp Neuropsychol. 1987;9(3):268.

[28] McCormick BJJ, Richard SA, Caulfield LE, Pendergast LL, Seidman JC, Koshy B, et al. Early life child micronutrient status, maternal reasoning, and a nurturing household environment have persistent influences on child cognitive development at age 5 years: results from MAL-ED. J Nutr. 2019;149(8):1460–9. doi: 10.1093/jn/nxz055 31162601 PMC6686051

[29] Rogawski McQuade ET, Scharf RJ, Svensen E, Huggins A, Maphula A, Bayo E, et al. Impact of Shigella infections and inflammation early in life on child growth and school-aged cognitive outcomes: Findings from three birth cohorts over eight years. PLoS Negl Trop Dis. 2022;16(9):e0010722. doi: 10.1371/journal.pntd.0010722 36149931 PMC9534434

[30] Prado EL, Larson LM, Cox K, Bettencourt K, Kubes JN, Shankar AH. Do effects of early life interventions on linear growth correspond to effects on neurobehavioural development? A systematic review and meta-analysis. Lancet Glob Health. 2019;7(10):e1398–413. doi: 10.1016/S2214-109X(19)30361-4 31537370

[31] Suryawan A, Jalaludin MY, Poh BK, Sanusi R, Tan VMH, Geurts JM, et al. Malnutrition in early life and its neurodevelopmental and cognitive consequences: a scoping review. Nutr Res Rev. 2022;35(1):136–49. doi: 10.1017/S0954422421000159 34100353

[32] Yousafzai AK, Rasheed MA, Rizvi A, Armstrong R, Bhutta ZA. Effect of integrated responsive stimulation and nutrition interventions in the Lady Health Worker programme in Pakistan on child development, growth, and health outcomes: a cluster-randomised factorial effectiveness trial. Lancet. 2014;384(9950):1282–93. doi: 10.1016/S0140-6736(14)60455-4 24947106

[33] Scharf RJ, Rogawski McQuade ET, Svensen E, Huggins A, Maphula A, Bayo E, et al. Early-life enteric pathogen exposure, socioeconomic status, and school-age cognitive outcomes. Am J Trop Med Hyg. 2023;109(2):436–422. 37536666 10.4269/ajtmh.22-0584 PMC10397442

[34] Westreich D, Greenland S. The table 2 fallacy: presenting and interpreting confounder and modifier coefficients. Am J Epidemiol. 2013;177(4):292–8. doi: 10.1093/aje/kws412 23371353 PMC3626058

