# Supplementary material for: Growth and cognition over eight years in three sites of the MAL-ED cohort study
Source: PLOS Glob Public Health. 2026 Sep 11;6(9):e0007217. doi: 10.1371/journal.pgph.0007217 (PMC13567774; doi:10.1371/journal.pgph.0007217)
Supplement: Supplementary file 1 [file pgph.0007217.s001.docx]

**S1 Table.** Comparison of characteristics between the 451 children assessed at 6-8 years of age and the 611 children in Brazil, South Africa, and Tanzania who completed the original MAL-ED study with follow-up to 2 years of age.

|  | Assessed at 6-8 years of age  (n=451) | Follow-up to 2 years of age  (n=611) |
| --- | --- | --- |
|  | Median (IQR). Median (IQR) | |
| Sociodemographic characteristics |  |  |
| Female sex (n; %) | 219 (48.6) | 297 (48.6) |
| Socioeconomic status | 0.72 (0.27, 0.84) | 0.73 (0.28, 0.84) |
| Percent days exclusively breastfed < 6 mo. | 28 (15, 46) | 28 (15, 45) |
| Maternal education (years) | 8 (7, 11) | 8 (7, 11) |
| Maternal height (cm) | 157 (152, 161) | 157 (152, 161) |
| Anthropometry |  |  |
| Enrollment weight-for-age z-score | -0.20 (-0.83, 0.43) | -0.18 (-0.80, 0.40) |
| Enrollment length-for-age z-score | -0.79 (-1.44, -0.19) | -0.81 (-1.45, -0.19) |
| 2 year length-for-age z-score | -1.73 (-2.60, -0.74) | -1.62 (-2.60, -0.61) |
|  |  |  |
|  |  |  |
|  |  |  |
|  |  |  |
|  |  |  |
|  |  |  |
|  |  |  |
|  |  |  |
|  |  |  |
|  |  |  |

*Missing for 7 children in Brazil, 5 children in South Africa, and 11 children in Tanzania

IQR = interquartile range
